# Switching to Aflibercept in Diabetic Macular Edema Not Responding to Ranibizumab and/or Intravitreal Dexamethasone Implant

**DOI:** 10.1155/2017/8035013

**Published:** 2017-08-16

**Authors:** Antoine Herbaut, Franck Fajnkuchen, Lise Qu-Knafo, Sylvia Nghiem-Buffet, Bahram Bodaghi, Audrey Giocanti-Auregan

**Affiliations:** ^1^Ophthalmology Department, Avicenne Hospital, DHU Vision and Handicaps, Paris XIII University, 125 rue de Stalingrad, 93000 Bobigny, France; ^2^Centre d'Imagerie et de Laser, 11 rue Antoine Bourdelle, Paris, France; ^3^Ophthalmology Department, Pitié Salpétrière Hospital, DHU Vision and Handicaps, Paris VI University, Paris, France

## Abstract

**Purpose:**

To assess short-term functional and anatomical outcomes of refractory diabetic macular edema (DME) following a switch from ranibizumab or dexamethasone to aflibercept.

**Methods:**

We included retrospectively eyes with persistent DME after at least 3 ranibizumab and/or one dexamethasone implant intravitreal injections (IVI). The primary endpoint was the mean change in visual acuity (VA) at month 6 (M6) after switching.

**Results:**

Twenty-five eyes were included. Before switching to aflibercept, 23 eyes received a median of 9.5 ranibizumab, and among them, 6 eyes received one dexamethasone implant after ranibizumab and 2 eyes received only one dexamethasone implant. Baseline VA, before any IVI, was 52.9 ± 16.5 letters, and preswitch VA was 57.1 ± 19.6 letters. The mean VA gain was +8 letters (*p* = 0.01) between preswitch and M6. The mean central retinal thickness was 470.8 ± 129.9 *μ*m before the switch and 303.3 ± 59.1 *μ*m at M6 (*p* = 0.001).

**Conclusion:**

Switching to aflibercept in refractory DME results in significant functional and anatomical improvement. The study was approved by the France Macula Federation ethical committee (FMF 2017-138).

## 1. Introduction

Diabetic macular edema (DME) is the leading cause of visual impairment in patients with diabetic retinopathy [1]. The prevalence of diabetes is increasing worldwide. In diabetic patients, the prevalence of DME reaches about 5% [2, 3]. The cost associated with visual disability and treatment is high and makes DME a global health issue.

DME is mainly due to an abnormal vascular permeability involving vascular endothelial growth factor (VEGF). Intravitreal anti-VEGF therapy is currently one of the treatments of DME along with corticosteroids: in 2012, ranibizumab has been approved by the Food & Drug Administration (FDA) following the RISE and RIDE studies [4]; then, aflibercept has been approved for the treatment of DME in 2014 following the VIVID and VISTA phase 3 clinical trials [5]. In France, aflibercept and dexamethasone are reimbursed by the healthcare system, respectively, since September and October 2015, while ranibizumab is reimbursed since 2012.

Thus, the first approved therapy with an intravitreally applied agent used in our practice in DME patients was ranibizumab because of its earlier availability. In our previously published real-life series of patients treated with ranibizumab, 18% did not respond to 3 intravitreal injections and showed a visual gain < 5 letters and an anatomical improvement in central retinal thickness (CRT) < 10% of the initial CRT [6]. Moreover, 26.5% of our patients never achieved a flat retina (<300 *μ*m) during the one-year follow-up. In these particular cases where ranibizumab is not effective, a switch to another treatment is needed. Clinicians may either change the pharmaceutical class and use dexamethasone implant, also approved in this indication [7], or switch to another anti-VEGF agent. Some studies [8–10] reported results supporting the efficacy of aflibercept after ranibizumab failure.

Unlike ranibizumab, aflibercept binds not only VEGF-A but also VEGF-B and placental growth factor (PlGF) [11]. This additional mechanism of action may also explain the possible efficacy of aflibercept after ranibizumab failure even if they belong to the same therapeutic class. A switch to another pharmaceutical class such as corticosteroids is also possible. Dexamethasone, the only corticosteroid reimbursed for the treatment of DME in France, has a nonspecific anti-VEGF effect associated with an anti-inflammatory effect and allows a continuous delivery over a few months.

The aim of this study was to assess the short-term outcomes following a switch from ranibizumab or dexamethasone therapy to aflibercept in the treatment of refractory DME.

## 2. Patients and Methods

A retrospective study was conducted in a tertiary center specialized in imaging and treatment of retinal diseases. All consecutive patients diagnosed with DME and treated with ranibizumab (0.5 mg) and/or dexamethasone (0.7 mg) and subsequently switched to aflibercept (2.0 mg) between June 2015 and August 2016 were included. Indications for switching to aflibercept included persistent DME under a previous well-conducted treatment, defined as no reduction, incomplete resolution (<10% improvement of central retinal thickness (CRT)), increase in central subfield thickening, or persistence of central cysts on SD-OCT, considered as significant by the investigator after a loading phase of at least 3 injections. The choice of switching was at the discretion of the investigator. Decision for switching to aflibercept after dexamethasone was taken when these same anatomical criteria were observed at the second visit (8 weeks) after at least one dexamethasone injection.

This study was conducted in accordance with the tenets of the Declaration of Helsinki, and an informed consent was obtained from all patients. Approval was obtained from the France Macula Federation ethical committee (FMF 2017-138).

Inclusion criteria were as follows: patients with type 1 or 2 diabetes, with persistent DME defined by a loss of the foveal pit, and a CRT > 300 *μ*m on SD-OCT (Cirrus 5000, ZEISS, Meditec) responsible for a loss of vision (preswitch visual acuity (VA)). Only patients who received at least the first 3 monthly aflibercept injections were included in the study.

Exclusion criteria were as follows: other ocular conditions impairing vision or complication of diabetic retinopathy (tractional retinal detachment, vitreous hemorrhage), fewer than three ranibizumab injections prior to the switch to aflibercept, and incomplete imaging or clinical data.

All patients underwent a complete baseline ophthalmological examination including VA on ETDRS chart, slit-lamp examination, fundus imaging, and SD-OCT. VA was measured monthly, and SD-OCT scans were assessed 4 weeks after the third injection and at month 6 of follow-up. Baseline VA was defined as the initial VA before any intravitreal treatment and preswitch VA as the VA just before switching to aflibercept. All patients were assessed every 4 weeks after the 3 initial aflibercept injections and treated on an as-needed, pro re nata (PRN), regimen in case of recurrence based on functional (VA < 78 letters or 20/32) and anatomical parameters (CRT > 300 *μ*m). Recurrence was considered as a loss of VA ≥ 5 letters between 2 consecutive visits or CRT > 300 *μ*m or a CRT increase > 10% or significant cyst reappearance at the discretion of the investigator.

SD-OCT scans were obtained using the Cirrus 5000 (ZEISS, Meditec) and were reviewed by the investigator to document the presence of intraretinal/subretinal fluid and decide if the patient needed additional injections.

The primary endpoint was the mean variation in VA between the preswitch time and month 6 (M6) after the switch.

Secondary endpoints were VA after 3 initial intravitreal injections of aflibercept (M3), CRT at 3 and 6 months, and number of injections.

Statistical analysis was performed using a *t*-test with graph pad software in the overall population after verification of a normal distribution. In the subanalyses and in small samples such as the dexamethasone group, for example, a nonparametric Mann–Whitney test was used in case of unpaired values or a Wilcoxon test in case of paired values. The results of VA and CRT are presented as mean ± SD. For small samples, the results were presented as median (min–max).

A *p* value <0.05 was considered statistically significant. Two subgroup analyses were performed: one analysis dividing final CRT into CRT < 300 *μ*m and CRT > 300 *μ*m and another dividing preswitch VA into VA < 20/40 (70 letters) and VA ≥ 20/40.

## 3. Results

Twenty-nine eyes were screened but the data at 6 months were not available for 4 eyes that were excluded for the following reasons: patient dropout (2 eyes) and 2 eyes underwent cataract surgery between 3 and 6 months after the first injection of aflibercept. Thus, 25 eyes of 21 patients met the inclusion criteria and were included. Patient mean age was 63.1 ± 10.8 years (range: 33–83 years). There was a slight female predominance with 13 women included (62%).

Baseline characteristics are presented in Table 1.

Before inclusion, 23 eyes received a mean number of 9 ± 4.6 (median: 9.5, range: 3–15 injections) ranibizumab injections, and among them, 6 eyes received a mean number of 1.5 (median: 1, range: 1–3 injections) dexamethasone implants following ranibizumab treatment. Two eyes received only one dexamethasone implant before switching to aflibercept. The mean follow-up duration was 2.2 ± 0.2 (1.9–2.6) months at M3 and 5.7 ± 0.5 (5. 2–7) months at M6 after the first aflibercept injection.

### 3.1. Visual Outcomes after Switching to Aflibercept

**Figure 1:**
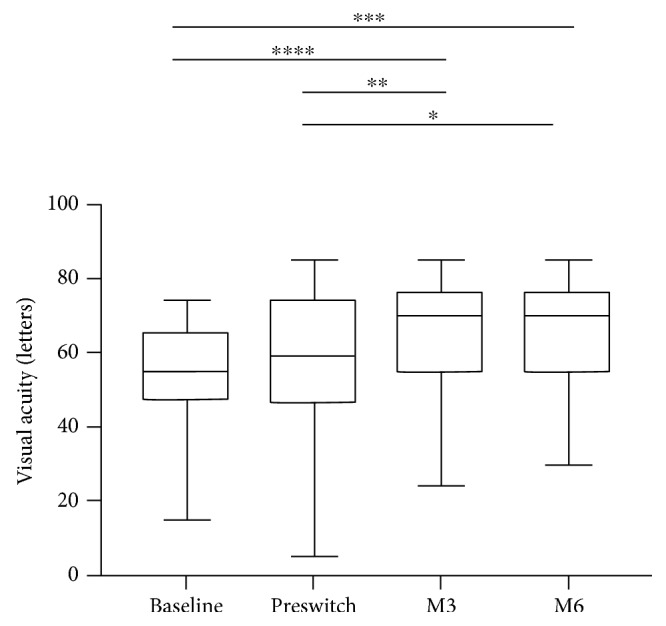
Change in visual acuity (ETDRS chart) over 6 months of follow-up after switch to aflibercept. Box plots representing at each time point the distribution of data from bottom to top: minimum, first quartile, median, third quartile, and maximum. *p* values were obtained after a paired parametric *t*-test after verification of the normal distribution. ^∗^<0.05; ^∗∗^<0.01; ^∗∗∗^<0.001; and ^∗∗∗∗^<0.0001.

The mean baseline VA before any intravitreal injection was of 52.9 ± 16.5 letters and the mean VA prior to the switch was of 57.1 ± 19.6 (+4.2 letters of visual gain). VA improved to 65.5 ± 16.4 letters (*p* = 0.006) and 65.1 ± 15.2 letters (*p* = 0.01) after 3 and 6 months of follow-up, respectively, corresponding to a mean VA change of +8.4 and +8 letters at 3 and 6 months (Table 2, Figure 1).

### 3.2. Anatomical Outcomes after Switching to Aflibercept

**Figure 2:**
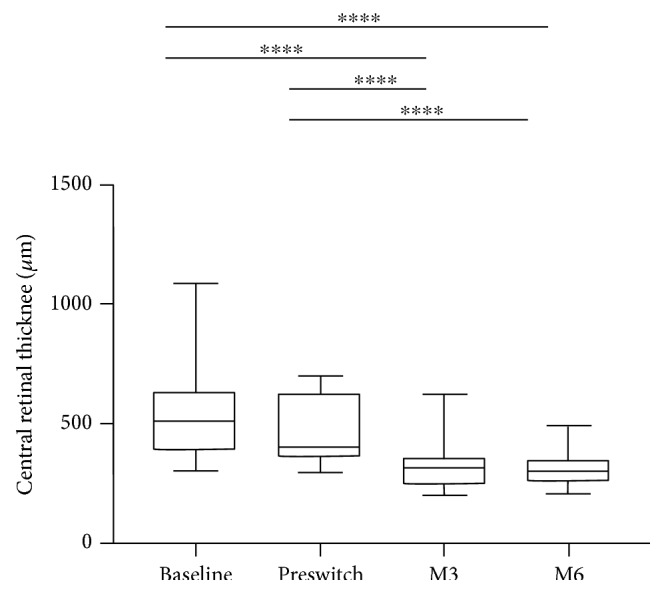
Change in central retinal thickness over 6 months of follow-up after switch to aflibercept. Box plots representing at each time point the distribution of data from bottom to top: minimum, first quartile, median, third quartile, and maximum. *p* values were obtained after a paired parametric *t*-test after verification of the normal distribution. ^∗∗∗∗^<0.0001.

The mean baseline CRT before any intravitreal injection was 532 ± 186.2 *μ*m, the mean CRT prior to the switch was 470.8 ± 129.9 *μ*m (−58.2 *μ*m), and a significant reduction to 315.6 ± 89.7 *μ*m (*p* = 0.001) and 303.3 ± 59.1 *μ*m (*p* = 0.001) was observed at 3 and 6 months, respectively, corresponding to a mean decrease of −155.2 ± 144.7 *μ*m and −167.5 ± 149.3 *μ*m, respectively, at 3 and 6 months (Table 3, Figure 2).

### 3.3. Subgroup Analysis

A subgroup analysis was performed to determine the percentage of eyes with a CRT < 300 *μ*m after switching to aflibercept and the impact on VA (Table 4).

After 6 months of follow-up, 60% of the eyes had a CRT < 300 *μ*m. Among them, the preswitch VA and CRT were 55.9 ± 20.4 letters and 497.4 ± 139.9 *μ*m, respectively, and they significantly improved to 67 ± 14.8 letters (*p* = 0.02) and 267.9 ± 28.7 *μ*m, corresponding to a mean VA change of +11.1 letters (Table 4).

At 6 months, 40% of the eyes still had a CRT > 300 *μ*m. In this group, the preswitch VA and CRT were 59 ± 19.1 letters and 460.2 ± 120.1 *μ*m and improved to 62.2 ± 16.3 letters (*p* = 0.44) and 356.3 ± 53.1 *μ*m at 6 months, corresponding to a mean VA gain of +3.2 letters.

The second subgroup analysis (Tables 5 and 6) was performed to determine the impact of the preswitch VA on the efficacy of aflibercept. Patients were divided into two groups: one with a preswitch VA < 70 letters (20/40 Snellen equivalent, low VA) and one with a preswitch VA ≥ 70 letters (high VA). The mean VA change was +10.6 ± 17.4 letters (*p* = 0.02) in the low VA group versus +2.4 ± 5.7 letters in the high VA group (*p* = 0.27). There was no significant difference in preswitch, M3, and M6 CRT between both groups (Table 6).

Eight eyes were previously treated with intravitreal dexamethasone implant before switching to aflibercept. Their median baseline VA was 45 (15–54) letters, and their preswitch median VA was 46.5 (5–74) letters. After switching, they improved their median VA to 54.5 (24–85) at M3 (*p* = 0.24 compared to preswitch VA) and 54.5 (24–85) at M6 (*p* = 0.34 compared to preswitch VA). Their mean VA change was +12.25 ± 22.4 (min: −17, max: +54) letters at M6.

Their median baseline CRT was 477 *μ*m (306–1088 *μ*m), and their preswitch median CRT was 390.5 *μ*m (327–696 *μ*m). After switching, they improved their median CRT to 284.5 *μ*m (204–623 *μ*m) at M3 (*p* = 0.03 compared to preswitch CRT) and 298.5 *μ*m (236–591 *μ*m) at M6 (*p* = 0.007 compared to preswitch CRT). Their mean CRT change was −170.5 ± 188 *μ*m (min: +91, max: −431) at M6.

The functional and anatomical outcomes among patients treated by ranibizumab monotherapy, dexamethasone monotherapy, or combined monotherapy before switching to aflibercept are presented in Table 7.

No serious adverse event following intravitreal injections was noted in this study.

## 4. Discussion

In this study, we showed a rapid anatomical and functional improvement in eyes with persistent DME that poorly responded to ranibizumab and/or dexamethasone after a switch to aflibercept.

Only a few studies have assessed the outcomes of a switch to aflibercept after chronic anti-VEGF therapy for persistent DME. In a prospective study, Wood et al. [9] have shown a significant anatomical improvement in 14 patients who switched to aflibercept after a single injection.

Another recent retrospective study [10] has shown a significant functional and anatomical improvement in 21 eyes after a switch to aflibercept. In this study, no fixed pattern was used for aflibercept treatment after the switch: a median number of 3 aflibercept injections was received during a mean follow-up of 5 months with an interval of 2.4 months between aflibercept injections.

In our study, when a switch was decided, we made the choice to prescribe a complete treatment protocol including 3 monthly aflibercept injections before patient assessment as we considered that the switch required a new loading phase of injections.

A more recent retrospective study [8] on this topic has shown a significant anatomical improvement and an overall trend to functional improvement after switching without reaching significance. However, half of the cohort did not attend the fourth visit after switching. In the 22 patients who attended the fourth visit after the switch, the VA was significantly increased. The authors have suggested that a longer follow-up after switching to aflibercept is necessary for a more accurate assessment of VA outcomes.

In our study, we found a functional and anatomical improvement 6 months after aflibercept switch. We assumed that this result could be due to our strict retreatment criteria mainly based on anatomical features instead of criteria based on the functional improvement only and to the systematic prescription of 3 monthly injections when the switch was decided. Indeed, we considered that a switch could be relevant even when the vision was improved although some fluid was still present in the retina. This assumption was confirmed, in particular in patients with a final CRT < 300 *μ*m; in addition to an initial visual gain of 5.2 letters between the baseline and the preswitch time, their VA improved by 11.1 more letters (*p* = 0.02) after the switch to aflibercept.

A subgroup analysis was performed to determine the impact of the preswitch VA. We found a higher final VA change of +10.6 letters when the preswitch VA was <70 letters, but with a lower final VA of 58 letters at 6 months versus a gain of only +2.4 letters in the group with a preswitch VA ≥ 70 letters, with a much higher final VA of 80.1 letters at 6 months, without any difference in CRT between both groups at preswitch, M3, and M6.

The slight visual gain in the group with the highest preswitch VA could be explained by the ceiling effect [12]. It is known that one of the good predictive factors of DME treatment is the baseline VA: the higher the baseline VA is, the better the final VA will be. However, our study showed that it is also important to switch when the preswitch VA is good, because the better the preswitch VA was, the better the final VA was in our patients.

However, our study was one of the first “real-life” study assessing the switch to aflibercept in DME resistant to ranibizumab at the dose of 0.5 mg. Similarly, for instance, Rahimy et al. [8] have explored the switch to aflibercept after treatment with 0.3 mg ranibizumab or bevacizumab.

Even with a higher dose of ranibizumab, 0.5 mg versus 0.3 mg, DME was still persistent in our series. After the switch to aflibercept and 6 months of follow-up, 60% of the eyes had a CRT < 300 *μ*m. The functional response was particularly important in these patients with a mean VA change of +11.1 letters. This study also confirmed that the treatment switch could improve anatomical and functional outcomes in patients with fluid persistence.

Switching to dexamethasone implant has shown good functional and anatomical outcomes after ranibizumab failure in DME treatment [13–15]. Here, in case of dexamethasone treatment failure (8 eyes), the VA gain was +12.25 letters at 6 months. Our study was the first, to the best of our knowledge, to analyze a switch from dexamethasone to aflibercept.

Our real-life short-term results are consistent with those of the VIVID and VISTA studies [16], with a mean VA before aflibercept injections of 57 letters versus about 59 letters in the VIVID and VISTA studies and a visual gain of +8 letters in our study versus between +8.5 and +11 letters in the VIVID and VISTA studies at 6 months.

In case of drug switch in DME treatment, it is easy to understand that a switch from corticosteroids, when ineffective, to anti-VEGF may improve CRT and VA by restoring the inner blood-retinal barrier through the differential effects of these various treatment classes. However, in cases of switch from ranibizumab to aflibercept, two anti-VEGF agents, the efficacy of aflibercept could be due either to a switch effect in case of autoantibody development to prior anti-VEGF therapy [17, 18] or to the different targets of both drugs. Indeed, ranibizumab only binds free VEGF-A leading to VEGFR2 inhibition only, while aflibercept binds VEGF-A, PlGF, and VEGF-B leading to VEGFR1 and 2 inhibition [11]. This differential pathophysiological effect could explain the effect of aflibercept after ranibizumab failure. However, no study of a switch assessing the opposite pattern is currently available (switch to ranibizumab and/or intravitreal dexamethasone implant in DME not responding to aflibercept) to confirm this assumption.

Our study is limited by its retrospective design, the absence of a control group (ranibizumab monotherapy, dexamethasone monotherapy, or combination of both) to compare outcomes of eyes not switched to aflibercept and receiving their initial treatment for an extended period of time. The follow-up of 6 months was not intended to observe the long-term effect of the treatment but mainly to confirm the efficacy of aflibercept treatment in case of failure of other therapies in DME.

In conclusion, despite its limitations, this study provides a potentially useful clinical insight into DME not responding to ranibizumab and/or dexamethasone in a real-life setting. Our results supports early DME treatment switch before patients experience a severe vision loss when the first therapy is not effective, since 60% of our patients achieved a complete fluid resolution and a good visual improvement.

## Figures and Tables

**Table 1:** Patient baseline demographics and clinical characteristics.

		Patient demographics and clinical characteristics (*n* = 21 patients, 25 eyes)
Gender		13 female/8 male
Age (years)	Median (min–max)	64 (33–83)
Diabetes	Type 1, *n* (%)	2 (9.6)
	Type 2, *n* (%)	19 (90.4)
Insulin/ODT/ODT + insulin (*n*)		12/3/6
HbA1c levels (%)	Median (min–max)	8.3 (7.5–10.7)
High blood pressure, *n* (%)		18 (78.3)
Pan retinal photocoagulation, *n* (%)		20 (75), 2 ongoing
Lens Status, *n* (%)	Phakic	12 (48)
	IOL	13 (52)
Ranibizumab (*n* = 23)	Median number of injections (min–max)	9.5 (3–15)
Ozurdex only (*n* = 2)	Median number of dexamethasone injections	1
Ranibizumab + Ozurdex (*n* = 6)	Median number of dexamethasone injections (min–max)	1 (1–3)
Macular laser history (*n*)		2

ODT: oral diabetes treatment.

**Table 2:** Functional outcomes: visual acuity before any intravitreal injection (baseline) and before and after switch to aflibercept at M3 and M6.

	Baseline	Preswitch	M3 postswitch	M6 postswitch
Number of eyes	25	25	25	25
Mean letter score (SD)	52.9 (16.5)	57.1 (19.6)	65.5 (16.4)	65.1 (15.2)
*F*	2.914			
*p* value (ANOVA test)	0.04^∗^			
Mean VA change from preswitch (SD)			8.4 (14.1)	8 (15.1)
*p* value (compared to preswitch VA)			0.006^∗∗^	0.01^∗^
Mean VA change from baseline (SD)		4.2 (3.3)	12.6 (11.6)	12.2
*p* value (compared to preswitch VA)		0.34	<0.0001^∗∗∗∗^	0.0003^∗∗∗^

*F*: result of variance test (ordinary one-way ANOVA). Except for ANOVA test, *p* values were obtained after a paired parametric *t*-test after verification of the normal distribution. ^∗^<0.05; ^∗∗^<0.01; ^∗∗∗^<0.001; and ^∗∗∗∗^<0.0001.

**Table 3:** Anatomical outcomes: central retinal thickness before any intravitreal injection (baseline) and before and after switch to aflibercept at M3 and M6.

	Baseline	Preswitch	M3 postswitch	M6 postswitch
Number of eyes	25	25	25	25
Mean CRT in *μ*m (SD)	532 (186.2)	470.8 (129.9)	315.6 (89.7)	303.3 (59.1)
*F*	21.47			
*p* value (ANOVA test)	0.0013^∗∗^			
Mean CRT change from preswitch in *μ*m (SD)			−155.2 (144.7)	−167.5 (149.3)
*p* value (compared to preswitch CRT)			<0.0001^∗∗∗∗^	<0.0001^∗∗∗∗^
Mean CRT change from baseline in *μ*m (SD)		−61.2 (176)	−216.4 (226.1)	−228.7 (212.2)
*p* value (compared to baseline CRT)		0.07	<0.0001^∗∗∗∗^	<0.0001^∗∗∗∗^

CRT: central retinal thickness; *F*: result of variance test (ordinary one-way ANOVA). Except for ANOVA test, *p* values were obtained after a paired parametric *t*-test after verification that the distribution was normal. ^∗∗^<0.01 and ^∗∗∗∗^<0.0001.

**Table 4:** Subgroup analysis according to CRT < 300 or ≥300 *μ*m at M6.

Final CRT (M6)			
	CRT < 300 microns (group 1)	CRT > 300 microns (group 2)	*p* value^a^ (comparison between groups 1 and 2)
Number of eyes	15	10	
Mean letter score at baseline prior to any injection (SD)	50.7 (17.3)	55.7 (15.9)	0.48
Mean letter score preswitch (SD)	55.9 (20.4)	59 (19.1)	0.7
Mean letter score postswitch M3 (SD)	67 (14)	63.4 (20.1)	1
Mean letter score postswitch M6 (SD)	67 (14.8)	62.2 (16.3)	0.45
Mean VA change from preswitch (SD)	11.1 (16.2)	3.2 (12.5)	0.2
F	7.38	0.96	
*p* value (ANOVA test)	0.003^∗∗^	0.4	
*p* value^b^ (comparison between VA preswitch and M6 within each group)	0.02^∗^	0.44	

ANOVA test was performed to assess significance between VA at baseline, preswitch, M3, and M6 after switch to aflibercept within each group. ^a^*p* values were obtained after an unpaired nonparametric Mann–Whitney test between groups 1 and 2 at each time point. ^b^*p* values were obtained after a paired nonparametric Wilcoxon test between preswitch VA and VA at M6. VA: visual acuity. ^∗^<0.05 and ^∗∗^<0.01.

**Table 5:** Subgroup analysis of the impact of the preswitch VA (< or ≥70 letters) on VA.

	Preswitch visual acuity		
	VA < 70 letters (group 1)	VA ≥ 70 letters (group 2)	*p* value^a^ (comparison between groups 1 and 2)
Number of eyes	17	8	
Mean letter score at baseline prior to any injection (SD)	48.1 (17.2)	63.9 (7.9)	0.007^∗∗^
Mean letter score preswitch (SD)	47.4 (15.8)	77.7 (5)	0.001^∗∗∗^
Mean letter score postswitch M3 (SD)	59 (15.8)	79.4 (5.3)	0.001^∗∗∗^
Mean letter score postswitch M6 (SD)	58 (13)	80.1 (5.6)	0.001^∗∗∗^
Mean VA change from preswitch (SD)	10.6 (17.4)	2.4 (5.7)	0.2
*F*	5.6	15.37	
*p* value (ANOVA test)	0.004^∗∗^	0.03^∗^	
*p* value^b^ (comparison between VA preswitch and M6 within each group)	0.02^∗^	0.5	

ANOVA test was performed to assess significance between VA at baseline, preswitch, M3, and M6 after switch to aflibercept within each group. ^a^*p* values were obtained after an unpaired nonparametric Mann–Whitney test between groups 1 and 2 at each time point. ^b^*p* values were obtained after an paired nonparametric Wilcoxon test between preswitch VA and VA at M6. VA: visual acuity. ^∗^<0.05; ^∗∗^<0.01; and ^∗∗∗^<0.001.

**Table 6:** Subgroup analysis of the impact of the preswitch VA (< or ≥70 letters) on CRT.

	Preswitch visual acuity		
	VA < 70 letters (group 1)	VA ≥ 70 letters (group 2)	*p* value^a^ (comparison between groups 1 and 2)
Number of eyes	17	8	
Mean CRT in *μ*m at baseline prior to any injection (SD)	582.7 (201.3)	430.5 (96.8)	0.03^∗^
Mean CRT in *μ*m preswitch (SD)	495.2 (142.7)	418.9 (82)	0.28
Mean CRT postswitch M3 (SD)	312.7 (106)	321.6 (40.6)	0.47
Mean CRT postswitch M6 (SD)	301.8 (69.1)	306.4 (32.5)	0.62
F	18.21	9.42	
*p* value (ANOVA test)	<0.0001^∗∗∗∗^	0.003^∗∗^	
*p* value^b^ (comparison between CRT preswitch and M6 within each group)	0.0002^∗∗∗^	0.008^∗∗^	

ANOVA test was performed to assess significance between CRT at baseline, preswitch, M3, and M6 after switch to aflibercept within each group. ^a^*p* values were obtained after an unpaired nonparametric Mann–Whitney test between groups 1 and 2 at each time point. ^b^*p* values were obtained after a paired nonparametric Wilcoxon test between preswitch CRT and CRT at M6 within each group. VA: visual acuity; CRT: central retinal thickness. ^∗^<0.05; ^∗∗^<0.01; ^∗∗∗^<0.001; and ^∗∗∗∗^<0.0001.

**Table 7:** Subanalysis assessing functional and anatomical outcomes depending on treatment received before switch (ranibizumab monotherapy, dexamethasone monotherapy, or combined therapy).

	*N*	Baseline	Preswitch	M3	M6	*F*	*p* value (ANOVA test)
*Visual acuity (letters on ETDRS chart)*							
Ranibizumab monotherapy median (min–max)	17	59 (15–74)	65 (37–85)	74 (35–85)	70 (50–85)	9.747	0.0002^∗∗∗^
Dexamethasone monotherapy median (min–max)	2	64 (54–74)	60 (46–74)	60 (35–85)	63.5 (44–83)	NA	NA
Combined therapy median (min–max)	6	39.5 (15–50)	43.5 (5–53)	54.5 (24–74)	54.5 (35–74)	2.5	0.15
*p* value^a^ (comparison to baseline VA)			0.09	0.0002^∗∗∗^	0.0035^∗∗^		
*p* value^b^ (comparison to preswitch VA)				0.004^∗∗^	0.03^∗^		
*Central retinal thickness (μm)*							
Ranibizumab monotherapy median (min–max)	17	513 (376–831)	476 (315–660)	324 (208–388)	296	24.75	0.0001^∗∗∗∗^
Dexamethasone monotherapy median (min–max)	2	373 (306–440)	531 (366–696)	468 (313–623)	314.5 (299–330)	NA	NA
Combined therapy median (min–max)	6	540 (350–1088)	390.5 (327–677)	251.5 (204–420)	272.5 (208–388)	5.019	0.053
*p* value^a^ (comparison to baseline CRT)			0.08	<0.0001^∗∗∗∗^	<0.0001^∗∗∗∗^		
*p* value^b^ (comparison to preswitch CRT)				<0.0001^∗∗∗∗^	<0.0001^∗∗∗∗^		

*F*: result of variance test (ordinary one-way ANOVA). ANOVA test was performed to assess significance between VA or CRT at baseline, preswitch, M3, and M6 after switch to aflibercept within each group except for dexamethasone group (*n* = 2). ^a^*p* values were obtained after a paired nonparametric Wilcoxon test in comparison to baseline VA or CRT within the ranibizumab group; ^b^*p* values were obtained after a paired nonparametric Wilcoxon test in comparison to preswitch VA or CRT within the ranibizumab group. VA: visual acuity; CRT: central retinal thickness. ^∗^<0.05; ^∗∗^<0.01; ^∗∗∗^<0.001; and ^∗∗∗∗^<0.0001.
